# 

**Published:** 1900-05

**Authors:** 


### BOOKS RECEIVED.

Progressive Medicine—Volume I., 1900. A Quarterly Digest of Advances. Discoveries and Improvements in the Medical and Surgical Sciences. Edited by Hobart Amory Hare, M. D., Professor of Therapeutics and Materia Medica in Jefferson Medical College of Philadelphia. Octavo, handsomely bound in cloth, 404 pages, 36 engravings and a colored plate. Philadelphia and New York: Lea Brothers & Co. Issued quarterly. Price, $10.00 per year.

Twentieth Century Practice. An International Encyclopedia of Modern Medical Science. By Leading Authorities of Europe and America. Edited by Thomas L Stedman, M. D, New York City. In twenty volumes. Volume xix. "Malaria and Microörganisms." New York: William Wood & Co. 1900.

Hartridge, Refraction. The Refraction of the Eye. A manual for students. By Gustavus Hartridge, F.R.C S., Senior Surgeon to the Royal Westminster Ophthalmic Hospital. Tenth edition. 1900. Illustrated, 12mo. P. Blakiston's Son & Co, 1012 Walnut St., Philadelphia, Pa. 1900. Price, $1.50 net.

The Anatomy of the Brain. A text-book for medical students. By Richard H. Whitehead, M. D. Professor of Anatomy in the University of North Carolina. Illustrated with forty-one engravings. 6¼x9½ inches. Pages, v—96. Extra vellum cloth, $1.00, net. Philadelphia. The F. A. Davis Co., Publishers, 1914-16 Cherry St. 1900.

Injuries to the Eye in their Medico-legal Aspect. By S. Baudry, M. D. Professor in the Faculty of Medicine, University of Lille, France, etc. Translated from the original by Alfred James Ostheimer, Jr., M.D., of Philadelphia. Pa. Revised and edited by Charles A. Oliver, A. M., M. D., Attending Surgeon to the Wills Eye Hospital; Ophthalmic Surgeon to the Philadelphia Hospital; Member of the American and French Ophthalmological Societies. etc. With an adaptation of the Medico-legal Chapter to the Courts of the United States of America. By Charles Sinkler, Esq., Member of the Philadelphia Bar. 5⅝x7⅞ inches. Pages, x—161 Extra cloth, $1.00, net. Philadelphia. The F. A. Davis Co., Publishers, 1914-16 Cherry St. 1900.

Elements of Clinical Bacteriology for Physicians and Students. By Dr. Ernst Levy, Professor in the University Strasburg, i. E., and Dr. Felix Klemperer, Privat docent in the University of Strasburg. i. E., second enlarged and revised edition. Authorised translation by Augustus A. Eshner, M. D., Professor of Clinical Medicine in the Philadelphia Polyclinic, etc. Philadelphia: W. B. Saunders. 1900. [Price, $2.50.]

A Hand-book for Nurses, By J. K. Watson, M.D., Edin. Late House-Surgeon Essex and Colchester Hospital; Assistant-House Surgeon Sheffield Royal Infirmary and Sheffield Royal Hospital. American edition, under supervision of A. A. Stevens, A. M., M. D, Professor of Pathology in the Women's College of Pennsylvania, etc. Philadelphia: W. B. Saunders. 1900. [Price, $1.50.]

A Cyclopedia of Practical Medicine and Surgery. A Concise Reference Book, alphabetically arranged, of Medicine, Surgery, Obstetrics, Materia Medica, Therapeutics and the various specialties, with particular reference to diagnosis and treatment. Compiled under the editorial supervision of George M Gould, M.D., and Walter L. Pyle, M.D. Philadelphia: P. Blakiston Son & Co. 1900.

